# Reducing V3 Antigenicity and Immunogenicity on Soluble, Native-Like HIV-1 Env SOSIP Trimers

**DOI:** 10.1128/JVI.00677-17

**Published:** 2017-07-12

**Authors:** Rajesh P. Ringe, Gabriel Ozorowski, Kimmo Rantalainen, Weston B. Struwe, Katie Matthews, Jonathan L. Torres, Anila Yasmeen, Christopher A. Cottrell, Thomas J. Ketas, Celia C. LaBranche, David C. Montefiori, Albert Cupo, Max Crispin, Ian A. Wilson, Andrew B. Ward, Rogier W. Sanders, P. J. Klasse, John P. Moore

**Affiliations:** a Department of Microbiology and Immunology, Weill Medical College of Cornell University, New York, New York, USA; b Department of Integrative Structural and Computational Biology, International AIDS Vaccine Initiative Neutralizing Antibody Center and the Collaboration for AIDS Vaccine Discovery, The Scripps Research Institute, La Jolla, California, USA; c Oxford Glycobiology Institute, Department of Biochemistry, University of Oxford, Oxford, United Kingdom; d Department of Immunology and Microbial Science, The Scripps Research Institute, La Jolla, California, USA; e Department of Surgery, Duke University Medical Center, Durham, North Carolina, USA; f Department of Medical Microbiology, Academic Medical Center, University of Amsterdam, Amsterdam, The Netherlands; Emory University

**Keywords:** HIV-1 vaccine, Env trimers, V3 region

## Abstract

Native-like trimers of the SOSIP design are being developed as immunogens in human immunodeficiency virus type 1 (HIV-1) vaccine development programs. These trimers display the epitopes for multiple broadly neutralizing antibodies (bNAbs) but can also expose binding sites for some types of nonneutralizing antibodies (non-NAbs). Among the latter are epitopes in the gp120 V3 region that are highly immunogenic when SOSIP trimers are evaluated in animal models. It is presently uncertain whether antibodies against V3 can interfere with the induction of NAbs, but there are good arguments in favor of suppressing such “off-target” immune responses. Accordingly, we have assessed how to minimize the exposure of V3 non-NAb epitopes and thereby reduce their immunogenicity by introducing *N*-glycans within the V3 region of BG505 SOSIP trimers. We found that inserting glycans at positions 306 and 314 (termed M1 and M7) markedly reduced V3 antigenicity while improving the presentation of trimer apex bNAb epitopes. Both added glycans were shown to be predominantly of the Man_6_GlcNAc_2_ form. The additional introduction of the E64K ground-state stabilizing substitution markedly reduced or ablated soluble CD4 (sCD4) induction of non-NAb epitopes in V3 and/or associated with the coreceptor binding site. When a V3 glycan- and E64K-modified trimer variant, BG505 SOSIP.664-E64K.M1M7, was tested in rabbits, V3 immunogenicity was eliminated while the autologous NAb response was unchanged.

**IMPORTANCE** Trimeric proteins are being developed for future HIV-1 vaccine trials in humans, with the goal of eliciting broadly active neutralizing antibodies (NAbs) that are active against a wide variety of circulating strains. In animal models, the present generation of native-like trimer immunogens, exemplified by the BG505 SOSIP.664 construct, induces narrow-specificity antibodies against the neutralization-resistant (tier-2), sequence-matched virus and more broadly active antibodies against sequence-divergent atypically neutralization-sensitive (tier-1) viruses. A concern in the trimer immunogen design field has been whether the latter off-target antibodies might interfere with the induction of the more desired responses to tier-2 epitopes. Here, we have inserted two glycans into the dominant site for tier-1 NAbs, the gp120 V3 region, to block the induction of off-target antibodies. We characterized the new trimers, tested them as immunogens in rabbits, and found that the blocking glycans eliminated the induction of tier-1 NAbs to V3-epitopes.

## INTRODUCTION

The human immunodeficiency virus type 1 (HIV-1) envelope glycoprotein trimer is a key element of vaccine development strategies aimed at inducing neutralizing antibodies (NAbs) (1–9). A now widely used immunogen design platform involves SOSIP trimers, which have been engineered for increased Env stability and can be produced and purified in practical quantities (1–3, 5, 6, 10–12). SOSIP trimers of multiple subdesigns and genotypes have been shown to mimic native, virion-associated trimers both antigenically and structurally, including by displaying the epitopes for many different broadly neutralizing antibodies (bNAbs) (1–3, 5, 6, 9, 11, 13–20). However, SOSIP trimers can also expose epitopes for some nonneutralizing antibodies (non-NAbs), particularly those associated with the V3 region of gp120. The extent of V3 exposure *in vitro* varies with the assay used to measure antibody binding and is far more pronounced in a capture enzyme-linked immunosorbent assay (ELISA) than in other methods, such as surface plasmon resonance (SPR) or negative stain electron microscopy (NS-EM) (5, 21). Of greater relevance is that V3 is clearly immunogenic when SOSIP trimers are tested as immunogens in animal models, with anti-V3 antibodies dominating the neutralization of tier-1 viruses (6, 15, 22, 23). In the same experiments, antibodies able to neutralize the autologous tier-2 viruses are induced, which is the more desired response (6, 15, 22, 23). A key question is whether the anti-V3 response is an irrelevant side effect of SOSIP trimer immunogenicity or whether it could, under certain circumstances, be an immunological distraction that compromises the induction of autologous or heterologous tier-2 NAbs.

The processes by which initial antibody responses can be driven to evolve toward bNAbs are likely to be highly complex and to require the sequential use of more than one immunogen. Arguments can be made that “off-target” antibody responses could interfere with the ones that are needed, for example, via immunogen complexing and sequestration or epitope competition events within germinal centers (24). For example, naive B cells with specificity for non-NAb epitopes such as V3 have been shown to outcompete naive B cells for bNAb epitopes (25). It is prudent, therefore, to explore ways to reduce the immunogenicity of V3 and other non-NAb epitopes on SOSIP trimers so as to focus the immune response elsewhere. We have already shown that the introduction of two sequence changes in the C1 (at residue 64 or 66) and V3 (at residue 316) regions of the prototypic SOSIP.664 trimer design can further stabilize the resulting SOSIP.v4.1 or SOSIP.v4.2 variants *in vitro* and reduce the immunogenicity of V3 in rabbits by severalfold. Nonetheless, the immunized rabbits still produced some anti-V3 antibodies that neutralized tier-1 viruses (15). An alternative BG505 SOSIP trimer design strategy led to broadly similar findings in guinea pigs (23). In neither study was the reduction in the tier-1 NAb response accompanied by a consistent increase in the autologous tier-1 NAb titers (15, 23).

Here, we have further addressed the question of V3 antigenicity and immunogenicity on SOSIP trimers. Our approach involved the addition of N-linked glycans at various positions within V3 to occlude the underlying non-NAb epitopes. The masking of potential epitopes by adding glycan sites is an established concept that has been used successfully to mask immunodominant epitopes in the V3 region of HIV-1 gp120 and in the variable head domain of the influenza virus hemagglutinin (26, 27). The same approach was also used to dampen the immune responses to heterologous trimerization domains added to subunit vaccine antigens (28). The outcome of our own studies was the identification of double-glycan mutant trimers of the BG505 and B41 genotypes that remained fully native-like but were no longer reactive with V3 non-NAbs *in vitro*. These glycan substitutions, at positions 306 (designated M1) and 314 (M7), could be combined with the E64K change in C1 that helps to prevent CD4-mediated induction of non-NAb epitopes associated with the coreceptor-binding site (15). When such a V3-masked and prefusion-stabilized trimer, BG505 SOSIP.664-E64K.M1M7, was tested as an immunogen in rabbits, NAb titers against various tier-1 viruses were reduced by 3- to 22-fold without compromising or improving the induction of autologous tier-2 NAbs. Additional analyses showed that the residual neutralization of the tier-1 viruses was not attributable to antibodies reactive with a cyclized BG505 V3 peptide. The V3-glycan masking method may be useful in designing more complex trimer-based strategies aimed at the eventual elicitation of bNAbs in humans.

## RESULTS

### Masking V3 epitopes via added glycans.

We assessed whether one or more epitope-masking glycans could be inserted into the V3 region of BG505 SOSIP.664 trimers. Because this project was initiated before the latest high-resolution structures of the trimer were available, we chose to insert glycans at multiple positions in V3 and to assess the properties of the resulting modified trimers empirically. Accordingly, potential N-linked glycosylation sites (PNGS) were introduced at 21 different positions in V3 via an NXT strategy; i.e., the first and third residues of each triplet were changed to N and T, respectively, to create a sequon (Table 1). The resulting single-glycan mutant proteins (D7324 tagged) were then transiently expressed in 293T cells, and the unpurified culture supernatants were used to assess Env antigenicity by capture ELISA (Table 2). The test panel included bNAbs to trimer-specific or trimer-sensitive epitopes (PG16, PGT145, and PGT151), as well as V3 non-NAbs (19b and 14e). Although nontrimer forms of Env with well-exposed V3 regions, such as dimers and monomers, contribute to the signals, this assay format is useful for screening multiple constructs and flagging the most promising variant for further evaluation. The outcome was identification of constructs M1 (S306N and R308T) and M7 (G314N and A316T) with reduced binding to V3 non-NAbs but with the epitopes for the trimer-specific bNAbs PG16, PGT145, and PGT151 retained (Table 2). As the M7 construct had a particularly promising overall antigenicity profile, we then combined the relevant G314N and A316T sequence changes with those present in the M1, M3, or M6 constructs to make three double-glycan mutants (M1M7, M3M7, and M6M7) that were similarly expressed and evaluated. Among them, the M1M7 mutant had the best antigenicity profile, with a marked reduction in V3 non-NAb binding combined with retention of trimer-specific bNAb epitopes (Table 2).

**TABLE 1:**
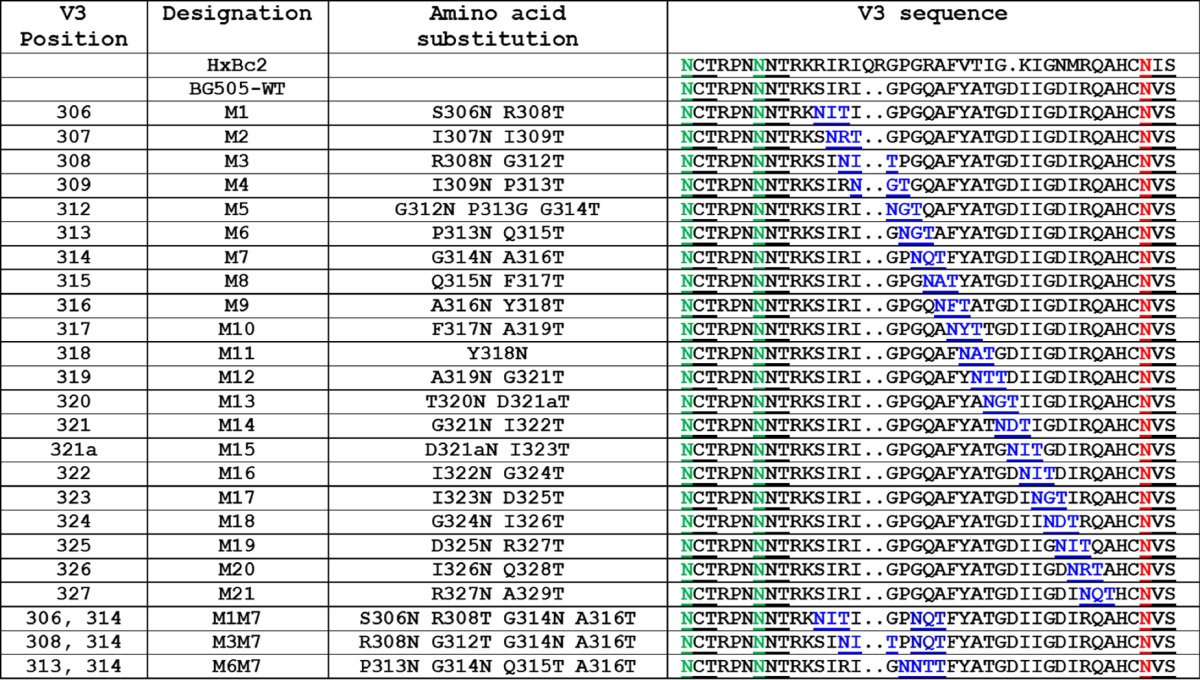
Design of BG505 SOSIP.664 V3 glycan mutants^*a*^

a The designations of the glycan mutants at V3 residues 306 to 327 are outlined in the first two columns. Residues were numbered using the HxBc2 system. Each construct was based on the BG505 SOSIP.664-D7324 background (designated BG505-WT, where WT is wild type). The specific substitutions in each mutant are listed in the third column, and the complete V3 sequence of the mutants, as well as of wild-type BG505-WT and HxBc2, are in the fourth column. The introduced glycan sites are shown in blue and underlined; the naturally present glycan sites at 295 and 301 are highlighted in green and underlined; the non-V3 but proximal glycan site at 332 is shown in red and underlined. Each of the introduced *N*-glycan motifs was of the NXT type, not NXS, to enhance the probability of glycan attachment (48–50). For construct M5, the P313G substitution was also made to increase the probability of glycan addition at position 312. For construct M15, inserting one additional residue at BG505 position 321 led to this residue being numbered 321a.

**TABLE 2:**
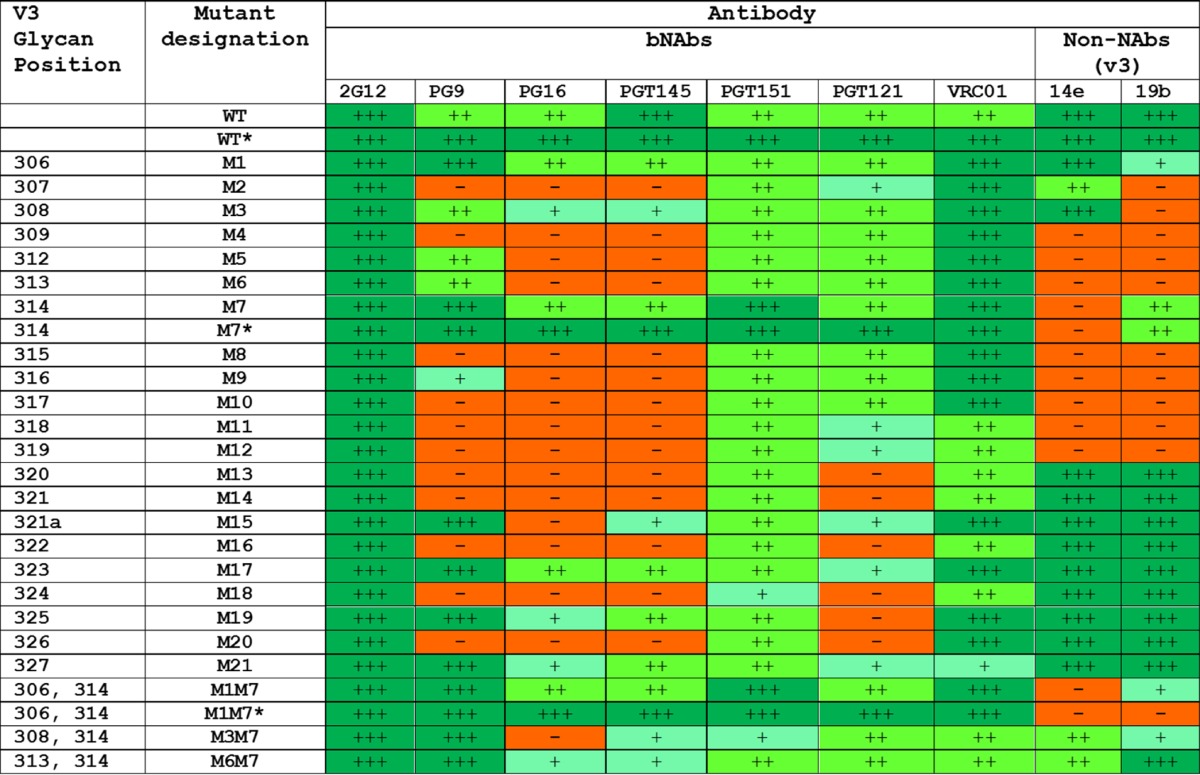
Antigenicity of BG505 SOSIP.664 V3 glycan mutants^*a*^

a The various mutants outlined in the first two columns contain one, or sometimes two, glycan sites inserted into various positions in V3 residue 306 to 327 (see Table 1 for additional details). Unless indicated by an asterisk (*), the antigenicity data were derived by D7324 capture ELISA using unpurified culture supernatants from 293T cell transient transfections. For mutants marked with an asterisk, D7324-tagged trimers based on the M7 and M1M7 constructs were also purified by the 2G12/SEC method, and the data (Fig. 1) are summarized here for comparison. For each antibody in the test panel, the magnitude of reactivity with the indicated Env proteins is recorded semiquantitatively on a scale from no binding (—) to strong binding (+++).

We studied the BG505 SOSIP.664-M7 and -M1M7 constructs in greater detail by expressing them in 293F cells and using a bNAb 2G12 affinity column and size exclusion chromatography (2G12/SEC) to purify the resulting D7324-tagged trimers and, for comparison, the parental BG505 SOSIP.664 trimer (Fig. 1). As assessed by capture ELISA, the antigenicity profiles of the M7 and M1M7 mutants were generally comparable to the profile of the parental trimer except for a substantial reduction in V3 non-NAb reactivity. More specifically, the M7 trimer was completely nonreactive with 14e but did still bind 19b and 39F, whereas the M1M7 trimer had no reactivity with 14e and 39F and bound 19b to only a minimal extent (Table 2; Fig. 1). The outcome of these exploratory studies was the adoption of the BG505 SOSIP.664-M1M7 design, which contains masking glycans at positions 306 and 314.

**FIG 1:**
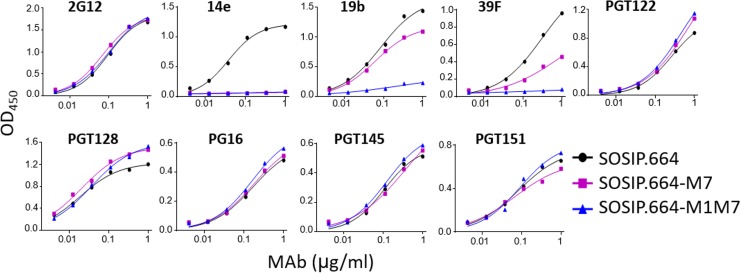
Comparative antigenicity of BG505 trimers assessed by ELISA. D7324-tagged BG505 SOSIP.664, -M7, and -M1M7 trimers were purified via the 2G12/SEC method and assessed for antibody binding by capture ELISA.

**FIG 2:**
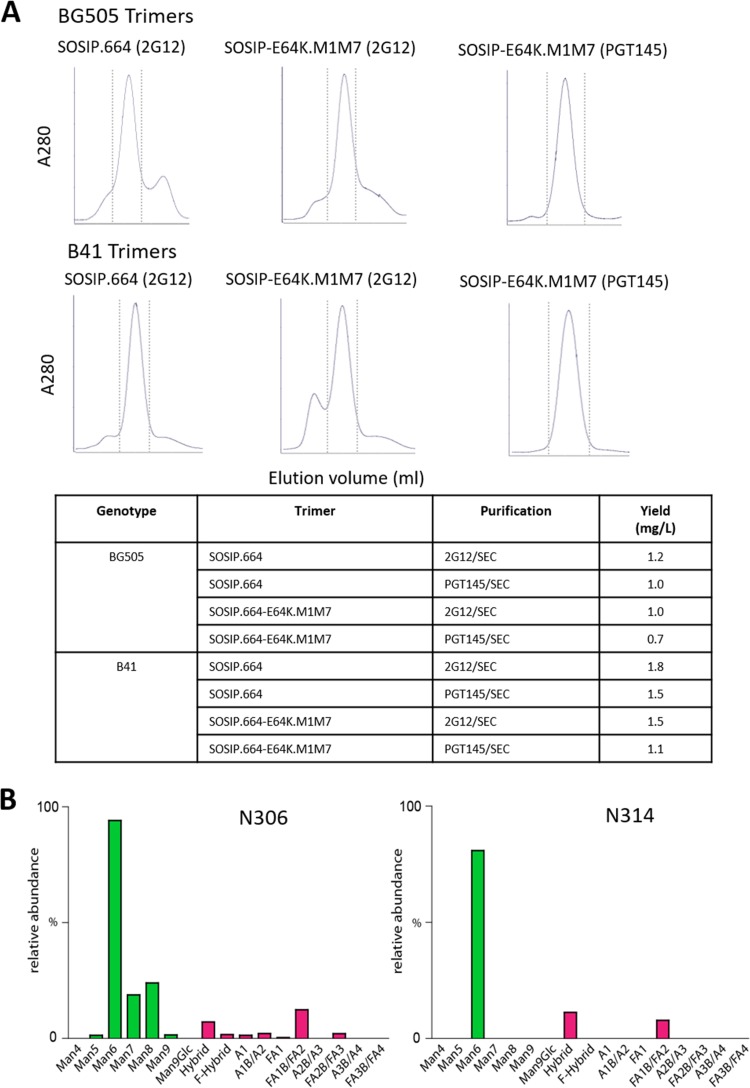
Glycan masking of V3 epitopes on BG505 and B41 SOSIP trimers. (A) SEC profiles of 2G12-purified or PGT145-purified, D7324-tagged BG505 or B41 SOSIP.664 and SOSIP.664-E64K.M1M7 Env proteins. The dotted vertical lines indicate the trimer peaks. The yields of trimers purified by the various methods are tabulated below the profiles. (B) Site-specific glycan analysis of the B41 SOSIP.664-E64K.M1M7 trimer. The glycan profiles for the corresponding BG505 trimer were similar (data not shown). The bars show the distribution and relative abundance of N-linked glycans displayed by type (green, oligomannose; pink, complex/hybrid). Glycans are categorized as oligomannose series (M5 to M9; Man_5_GlcNAc_2_ to Man_9_GlcNAc_2_), hybrids (H), and fucosylated hybrids (FH) and also by the number of branching antennae (A) of complex-type glycans. An, number (n) of antennae; B, bisected GlcNAc; F, presence of a core fucose.

The E64K change in the gp120 C1 domain has been shown to reduce the baseline and soluble CD4 (sCD4)-induced exposure of the coreceptor binding site and its associated non-NAb epitopes (15). We therefore introduced this change into the M1M7-modified trimers to make the SOSIP.664-E64K.M1M7 construct. We also produced the corresponding mutants of the clade B B41 SOSIP.664 trimer. The D7324-tagged versions of both genotypes of trimer variants were expressed by transient transfection of 293F cells and purified on either 2G12/SEC or PGT145/SEC columns. The SEC profiles were broadly similar for both methods, with a slightly lower content of nontrimer proteins in the PGT145-purified preparations (Fig. 2A). The overall yields of trimers were comparable for each genotype and purification method (within ±2-fold), implying that addition of the two V3 glycan sites did not adversely affect the overall biochemical properties of the trimers.

A site-specific analysis of the glycosylation of the B41 and BG505 SOSIP.664-E64K.M1M7 trimers, produced in 293F cells and purified via the PGT145/SEC method, showed that the introduced N306 (M1) and N314 (M7) sites were both modified by glycosylation. The dominant glycan present at both positions was Man_6_GlcNAc_2_ (Fig. 2B). The dominance of the Man_6_ glycan suggests that the presence of the neighboring, wild-type glycans impedes the further processing of the newly inserted ones (29, 30).

### Antigenicity and conformation of V3 glycan-modified trimers.

**FIG 3:**
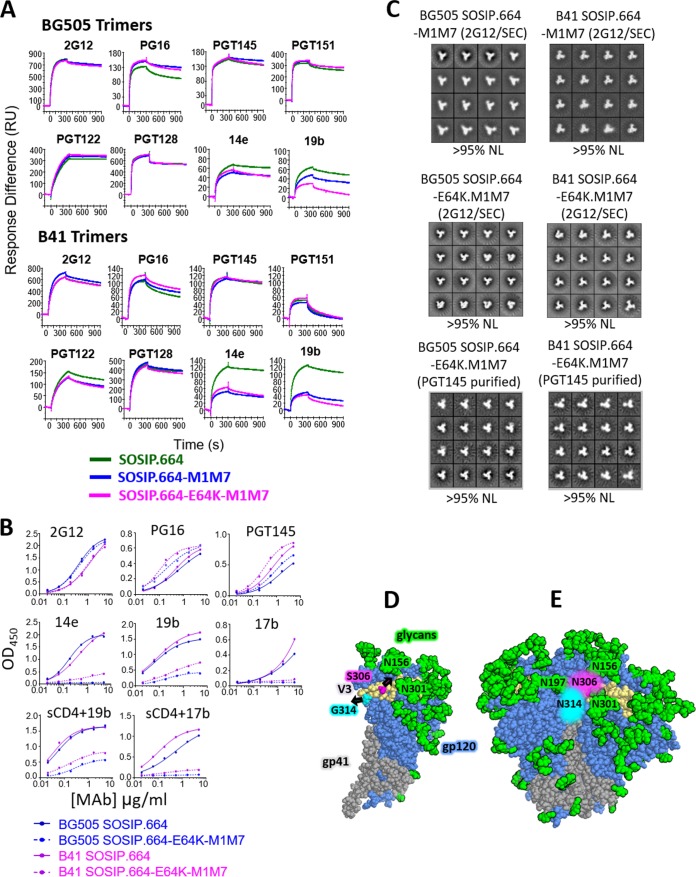
The V3 glycan-masked BG505 and B41 SOSIP trimers retain a native conformation as assessed by bNAb binding using SPR and ELISA. (A) The indicated PGT145/SEC-purified, His-tagged SOSIP.664, SOSIP.664-M1M7, and SOSIP.664-E64K.M1M7 trimers were analyzed by SPR. The test antibodies were used at 500 nM. The response difference (RU) is given on the *y* axis as a function of time (s) on the *x* axis. (B) ELISA of the same purified SOSIP.664 and SOSIP.664-E64K.M1M7 trimers used in the experiment shown in panel A to assess bNAb and non-NAb reactivity, with sCD4 also present when noted. The amount of each B41 and BG505 trimer comparator tested was adjusted to give equal binding of 2G12 bNAb for normalization purposes. (C) The indicated SOSIP.664-M1M7 (2G12/SEC-purified) and SOSIP.664-E64K.M1M7 (2G12/SEC or PGT145/SEC-purified) trimers, all D7324 tagged, were visualized by NS-EM. The percentages of the total trimers with native-like (NL) conformation are recorded below each collage. (D) Model of a single, wild-type BG505 gp120 protomer. The sites of glycan addition at residues S306 and G314 are shown in pale blue and magenta, respectively, with the geometrically favorable directions of the mutated asparagine side chains represented by arrows in the same colors. (E) Model of the BG505 SOSIP.664-E64K.M1M7 trimer. The estimated positions of inserted N306 (M1) and N314 (M7) glycans are shown in pale blue and magenta, respectively, with no indication of their detailed composition. In both panels D and E, the wild-type glycans are shown in green, with selected ones labeled, and both models are based on PDB accession number 5FYL.

An SPR analysis using PGT145-purified, His-tagged proteins showed that the double-glycan-modified BG505 and B41 trimers had almost unchanged 2G12 reactivities, which serves as a frame of reference for other epitopes (Fig. 3A). The SPR antigenicity profiles of the SOSIP.664, SOSIP.664-M1M7, and SOSIP.664-E64K.M1M7 variants of each genotype were broadly similar with respect to bNAbs against V3 glycan (2G12, PGT122, and PGT128), interface (PGT151), and quaternary apical (PG16 and PGT145) epitopes; if anything, the binding of PG16 was somewhat enhanced for the mutants, particularly for SOSIP.664-E64K.M1M7. In contrast, the binding of the 14e and 19b non-NAbs to their V3 epitopes was reduced for the SOSIP.664-M1M7 and SOSIP.664-E64K.M1M7 trimers compared with that of SOSIP.664. The differences in 14e binding were less marked for these glycan-modified BG505 trimers than for their B41 counterparts (Fig. 3A). However, we note that the unmodified BG505 SOSIP.664 trimers bound these V3 non-NAbs more weakly than B41 SOSIP.664, as shown here and in previous SPR assays (5, 21).

We also compared the antigenicity of the His-tagged BG505 and B41 SOSIP.664 and SOSIP.664-E64K.M1M7 trimers by capture ELISA (Fig. 3B). For both genotypes, binding of the PG16 and PGT145 bNAbs to the modified trimers was modestly improved, similar to the observations made with PG16 in the SPR analysis (Fig. 3A). Thus, the introduction of the V3 glycans certainly does not impair but, rather, modestly improves the presentation of the highly conformational bNAb epitopes at the trimer apex. The non-NAbs 14e, 19b, and 17b bound markedly less well. In addition, the ability of sCD4 to induce the exposure of the 19b and 17b non-NAb epitopes was greatly diminished or even fully ablated on the modified trimers (Fig. 3B).

NS-EM imaging of the BG505 and B41 SOSIP.664-M1M7 and SOSIP.664-E64K.M1M7 trimers confirmed that they were all fully native like (Fig. 3C). Clearly, the two new V3 glycans, with or without the additional ground state-stabilizing E64K change in C1, are fully consistent with the retention of an appropriate trimer conformation while greatly reducing or even ablating the presentation of non-NAb epitopes associated with V3 and the coreceptor binding site.

A model of the BG505 SOSIP.664-E64K.M1M7 trimer suggests that there is enough solvent-exposed area to accommodate both glycans near the respective mutation sites. The N306 glycan fills a gap between the existing N156 and N301 glycans but is constrained by the N197 glycan from a neighboring protomer (Fig. 3D and E). The new glycan at position N314 is more constrained than the one at N306 because of its location at the tip of V3. The most likely orientation of the N314 glycan on one gp120 protomer would result in a clash with the neighboring protomer (Fig. 3D and E). However, the substantial occupancy of the N314 site shown by the site-specific analysis (Fig. 2B) and the retention of native-like conformation as judged by NS-EM (Fig. 3C) together suggest that some conformational change must occur in this region of the M1M7 trimer. We speculate that accommodation of the N314 glycan must necessitate some rearrangement of the V3 region, at least at the tip, but in a manner whereby the closed conformation of the trimer apex is retained.

### Immunogenicity of BG505 SOSIP.664-E64K.M1M7 trimers in rabbits.

**FIG 4:**
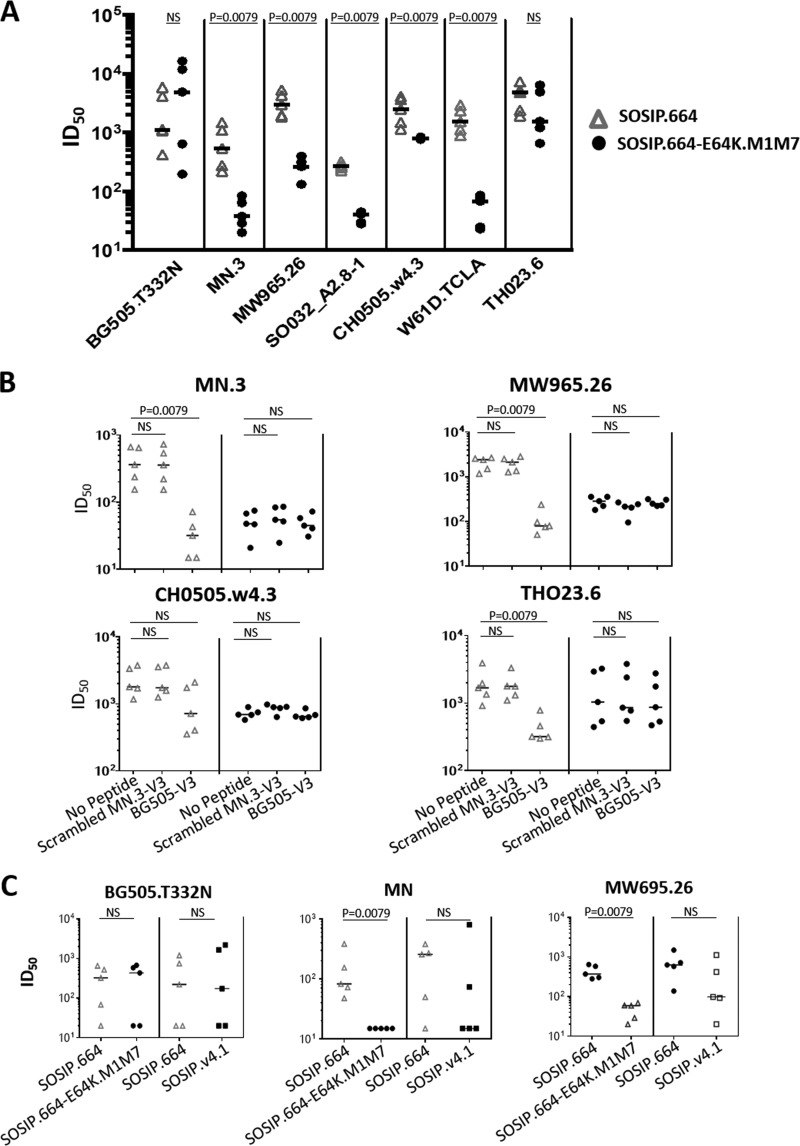
NAb responses to BG505 SOSIP.664 and SOSIP.664-E64K.M1M7 trimers in rabbits. (A) NAb titers of the two groups of rabbits are plotted for each test virus, all of which have the tier-1 neutralization phenotype except for the autologous BG505.T332N virus (tier-2). (B) The NAb titers for the same groups of rabbits against the indicated tier-1 viruses were measured after preincubation of the sera with or without the indicated cyclized V3 peptide. In both plots, each data point represents an individual rabbit, with the median value for each group of 5 indicated by the horizontal bar. Significant titer differences are indicated by the *P* values. (C) NAb titers for the autologous tier-2 virus BG505.T332N and the tier-1 viruses MN and MW965.26 are shown for the SOSIP.664 and SOSIP.664-E64K.M1M7 comparator groups described in the present study (left panels) and for the SOSIP.664 and SOSIP.v4.1 comparator groups described previously (right panels) (15). The data in panels A and B were generated at DUMC, and data in panel C were generated at WCMC. There was no overlap between the titer values in the two immunogen groups, and the *P* values from the nonparametric Mann-Whitney U test were identical and minimal (*P* = 0.0079).

To assess whether M1M7 glycans reduce the immunogenicity of V3 non-NAb epitopes *in vivo*, we immunized groups of five rabbits three times with the BG505 SOSIP.664-E64K.M1M7 trimers and SOSIP.664 trimers and quantified the NAb titers in serum from 2 weeks after the third immunization (Fig. 4A). The tier-2 autologous NAb titers were statistically indistinguishable for the two groups of rabbits (*P* = 0.69, NS). Thus, although the range of titers for the SOSIP.664 group was somewhat narrower than for SOSIP.664-E64K.M1M7, the titers for the individual animals fell within the same titer range. In marked contrast, NAb titers against the six tier-1 viruses were 3- to 22-fold lower for the SOSIP.664-E64K.M1M7 trimer group than for the SOSIP.664 group. The differences were significant for five of the viruses (*P* = 0.0079) but not for TH023.6 CRF01_AE (*P* = 0.30) (Fig. 4A).

In a TZM-bl cell assay at Weill Cornell Medical College (WCMC), none of the 10 sera neutralized any of the heterologous tier-2 viruses (clade A viruses 92UG037.8, Q23env17, and Q46envE2 and clade C virus 25710-2.43) at titers of >20. In a similar assay at Duke University Medical Center (DUMC), the same sera were tested against a panel of nine heterologous tier-2 viruses: Ce1176_A3 (clade C), 246-F3_C10_2 (clade A/C), CH119.10 (circulating recombinant form 07-BC), Ce703010217_B6 (clade A), CNE55 (circulating recombinant form 01_AE), 25710-2.43 (clade C), TRO.11 (clade B), BJOX002000.03.2 (circulating recombinant form 07_BC), and X1632-S2-B10 (clade B). Compared to a cutoff titer value of 20, only 2 of the 10 sera yielded any positive titers: one serum from the SOSIP.664 group neutralized 25710-2.43 (titer of 82), Ce1176_A3 (titer of 29), 246-F3_C10_2 (titer of 29), and CNE55 (titer of 31), and one serum from the SOSIP.664-E64K.M1M7 group neutralized 25710-2.43 (titer of 23). Thus, there is no evidence that suppressing the V3 response to SOSIP.664 trimers is sufficient to increase the breadth of the neutralization response against tier-2 viruses, which is consistent with earlier reports (15, 22, 23).

We used a cyclized BG505 V3 peptide as a soluble competitor in the neutralization assay to deplete V3-targeted antibodies active against tier-1 viruses. Previous studies indicated that this method reduced the tier-1 NAb response to SOSIP trimers but not the autologous tier-2 NAb response (6, 22). When the cyclized V3 peptide was mixed with sera from the BG505 SOSIP.664 trimer-immunized animals, the median titers against the MN.3, MW965.26, CH0505.w4.3, and TH023.6 tier-1 viruses were reduced by 11-, 30-, 2.5-, and 5.3-fold, respectively. A sequence-scrambled version of an MN.3 V3 peptide, used as a negative control, had no detectable effect (Fig. 4B). In contrast, the cyclized BG505 V3 peptide did not detectably reduce neutralization of any of the four tier-1 viruses by sera from the SOSIP.664-E64K.M1M7 trimer recipients (Fig. 4B). This outcome shows that the modifications made to this trimer ablate its ability to induce any antibodies that cross-react with the cyclized V3 peptide (Fig. 4B). For each virus, the residual level of tier-1 virus neutralization by the SOSIP.664 serum in the presence of the BG505 V3 peptide was similar to or lower than that mediated by the SOSIP.664-E64K.M1M7 serum. The residual neutralization mediated by the SOSIP.664 trimer serum in the presence of the V3 peptide, as well as by the SOSIP.664-E64K.M1M7 serum in the absence of the peptide, might be directed against other tier-1 epitopes such as those associated with the CD4 binding site (CD4bs) or CD4-induced (CD4i) sites. It is possible that antibodies to glycan-dependent V3 epitopes might also be induced by the modified trimer, but such epitopes on HIV-1 Env proteins are generally considered to be very poorly immunogenic and, hence, not likely to be generated in the context of this type of immunization procedure. We conclude, therefore, that adding the M1M7 glycans effectively eliminates the induction of cross-reactive, tier-1 NAbs by the V3 region of the modified trimer without compromising the ability of the trimers to induce autologous tier-2 NAbs to other epitopes.

Reductions in V3 immunogenicity were previously found when V3-stabilized BG505 SOSIP.v4.1 trimers were compared with SOSIP.664 trimers in rabbits (15). Here, we compared sera from that earlier rabbit experiment and the present one for their abilities to neutralize the MN.3 and MW965.26 tier-1 viruses in the same assay. Compared in each case to the corresponding SOSIP.664 trimer immunogen group, the tier-1 NAb titers were significantly reduced for the SOSIP.664-E64K.M1M7 group (*P* = 0.0079) but not for SOSIP.v4.1. This finding was made with both the MN.3 and MW965.26 test viruses. In contrast, there were no significant differences in the abilities of the four groups of sera to neutralize the autologous tier-2 virus BG505.T332N (Fig. 4C).

### Mapping the target for NAbs against the BG505.N332 autologous virus.

We have previously shown that a frequent, but not the only, target for the autologous NAb response in BG505 SOSIP.664 trimer-immunized rabbits is a hole in the glycan shield formed by the absence of glycans from positions 241 and 289 (3). Thus, sera from ∼60% of the immunized rabbits completely failed to neutralize BG505.T332N virus mutants with a glycan knocked in at position 241 and/or 289. The remaining sera usually also target this same glycan hole but in a way that is less sensitive to the knocked-in glycans, or they also recognize additional neutralization-relevant epitopes. The use of clones of the maternal MG505 virus, with and without specific sequence changes, provides additional information (3). To see whether the introduction of the M1M7 glycans had fundamentally changed the immunogenicity of the BG505 trimers, we used the same panel of mutant viruses to test the week 22 sera from both immunization groups (Table 3). Overall, we found no major difference between the SOSIP.664 and SOSIP.664-E64K.M1M7 immunogen groups. Some sera from these groups contained NAbs that targeted only the 241/289 glycan hole (e.g., sera 2111 and 2116), whereas the NAbs in the remaining sera were less affected by the presence of these knocked-in glycans (Table 3). This outcome is generally consistent with what we have previously reported for other sera from BG505 SOSIP.664 trimer-immunized rabbits (3).

**TABLE 3:**
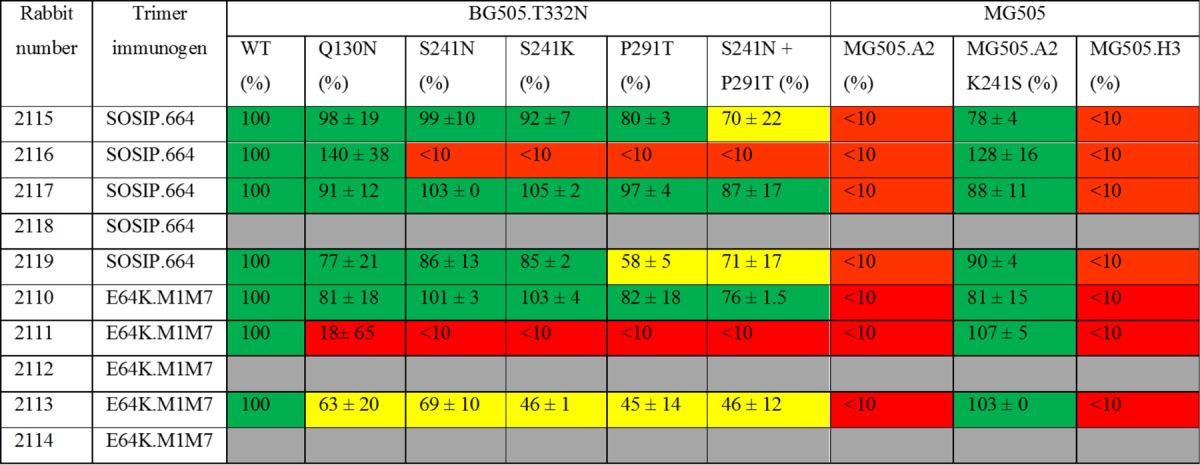
Neutralization of variant BG505 and MG505 viruses by rabbit sera^*a*^

a The rabbit sera tested were from week 22, which is 2 weeks after the third and final immunization with the BG505 SOSIP.664 or SOSIP.664-E64K.M1M7 trimer. The Q130N, S241N, and P291T changes introduce N-linked glycans at BG505.T332N virus positions 130, 241, and 289, respectively, in Env. The MG505.A2 and MG505.H3 viruses are different clones of the maternal MG505 isolate. The K241S substitution introduces a sequence change within the 241/289 glycan hole of the MG505.A2 clone. The values recorded for various mutant viruses are the percent neutralization values at a dilution of 1/50, relative to the BG505.T332N parental virus (labeled WT and defined as 100%), and are the averages of two or three replicates ± standard errors of the means. Red and yellow boxes indicate strong (>75%) and partial (25 to 75%) loss of neutralizing activity compared to that of the wild-type virus, respectively, with green boxes indicating no meaningful change. For rabbits 2118, 2112, and 2114, the titers against the wild-type virus were not high enough to allow mapping of the epitope (gray boxes).

## DISCUSSION

When native-like SOSIP trimers are used as immunogens in animals, they induce NAbs against both autologous tier-2 viruses and heterologous tier-1 viruses. These two responses are noncorrelated within individual animals and among test groups (3, 6, 15). Moreover, the tier-1 NAbs generally emerge relatively early in the immunization course (3, 6). The tier-1 NAbs are substantially attributable to V3 peptide-reactive antibodies, whereas the autologous tier-2 NAbs that have been mapped to date recognize more complex epitopes involving holes in the glycan shield (3). The latter type of response may be a necessary first step toward the development of bNAbs (6, 11, 24). It is not known whether off-target V3 antibodies interfere with the elicitation or evolution of bNAbs against the more relevant tier-2 epitopes, but there are various mechanisms by which this could happen, as discussed further below. Here, our goal was to identify ways to reduce the antigenicity of the V3 region on SOSIP trimers and to assess whether there was a concomitant reduction in V3-dependent non-NAbs or tier-1 NAbs when the modified trimers were used as immunogens.

The prototypic BG505 SOSIP.664 native-like trimer and others with similar *in vitro* properties expose multiple bNAb epitopes but very few for non-NAbs. Among the latter, V3 epitopes are the most prominently exposed, but the extent depends on the assay used to measure antibody binding. Thus, V3 non-NAb reactivity with SOSIP.664 trimers is much stronger in capture ELISAs than when measured by SPR or Octet (biolayer interferometry [BLI]) methods (5, 14). Moreover, V3 exposure is now considered to be a consequence of trimer breathing, i.e., reversible transitions between alternative conformations of the trimer that predominantly involve the V1V2 loop structure at the apex but that also affect the orientation and exposure of V3 (31, 32). Although what happens to trimers under *in vivo* conditions is not known, the exposure of highly immunogenic V3 epitopes elicits tier-1 NAb or non-NAb responses in rabbits, guinea pigs, and mice (3, 6, 11, 15, 22, 23, 33). Several methods to reduce the immunogenicity of the V3 region on BG505 SOSIP trimers have now been described (6, 11, 15, 22, 23). Thus, a point substitution, A316W, that helps lock the V3 region into the body of the trimer was made in concert with another in C1, either E64K or H66R, that reduces the spontaneous and CD4-driven exposure of CD4i non-NAb epitopes. The resulting BG505 SOSIP.v4.1 (A316W plus E64K change) and SOSIP.v4.2 (A316W plus H66R change) trimers were more stable, had superior antigenicity profiles, and elicited lower titers of V3-dependent tier-1 NAbs in rabbits than the SOSIP.664 prototype (15). Broadly similar reductions in V3 responses were achieved by complexing a BG505 SOSIP.664 trimer via a PGT145 Fab to occlude the V3 region (22) and by making several sequence changes that individually or collectively stabilize the trimer and reduce V3 exposure (23). However, in each of these studies, as here, the autologous tier-2 NAb responses to the variously modified trimers were generally similar to those elicited by the SOSIP.664 prototype (6, 11, 15, 22, 23).

The focus of this study was to assess whether the addition of masking glycans within V3 could further suppress the immunogenicity of this region of SOSIP trimers. There are precedents for the use of this method with earlier-generation Env proteins. Thus, the addition of glycans to the V3 region of a monomeric gp120 protein reduced the induction of anti-V3 antibodies in immunized guinea pigs while increasing the elicitation of anti-V1 antibodies (26). Otherwise-antigenic V3 epitopes were successfully masked when four glycans were introduced into this region of an uncleaved, nonnative YU2 gp140 protein (34). However, when wild-type and glycan-masked gp140s were tested in mice, the reduced immunogenicity of the glycan-masked V3 region did not divert the B cell response to other epitopes on the gp140 protein (34). The same glycan-immunosilencing method has also been applied successfully to other protein immunogens (27, 28).

After conducting exploratory studies on the BG505 SOSIP.664 construct, we identified residues 306 and 314 as positions where two glycans could be added without compromising the native-like conformation and bNAb epitope presentation of the resulting SOSIP.664-M1M7 trimers. A further improvement to antigenicity was achieved by the additional introduction of the E64K change in C1 that helps maintain the trimer in the prefusion ground state (15). The final glycan-modified BG505 and B41 trimers were fully native-like as judged by NS-EM, and site-specific glycan analysis showed that both of the inserted glycan sites were modified by glycosylation.

As noted earlier, this project was initiated without the guidance of high-resolution structural information. Had we in fact used the now available structures to design the mutants, we may have decided to not make the M7 and thence the M1M7 constructs that turned out to yield the most promising trimer variants. A model of the glycan-modified BG505 trimer shows that the N306 glycan fills a hole between two existing glycans at positions N156 and N301, while the N314 glycan at the V3 tip shields the remainder of V3. We do not know exactly how the N314 glycan is accommodated from the structural perspective, but it seems likely that some such process must take place to minimize a clash between the glycan on one protomer with the neighboring protomer. As residue 314 lies at the interprotomer interface, it might be anticipated that introducing a glycan into such an environment would create a steric clash with the adjacent protomer and destabilize the trimer. However, the antigenicity and low-resolution EM data together show that no such destabilization occurs. If and when a high-resolution structure of the M1M7 trimer is generated, we may better understand the orientation of the glycan-modified V3 region. Until then, our prevailing hypothesis is that the somewhat plastic trimer apex may open up sufficiently to the N314 glycan to be accommodated. Whatever static or dynamic events are taking place, the SOSIP.664-E64K.M1M7 trimers are fully native-like when viewed by NS-EM, and they retain (and even modestly improve) their presentation of bNAb epitopes, including the highly conformationally sensitive ones located at the trimer apex. In contrast, non-NAbs to V3 are minimally reactive or nonreactive with the glycan-modified trimers. The latter outcome is entirely consistent with the model of the modified trimer, which shows that very little of the surface of the V3 region is now accessible while the antigenically native, prefusion structure of Env is not disrupted.

A rabbit immunogenicity study showed that the glycan-modified SOSIP.664-E64K.M1M7 and parental SOSIP.664 trimers induced comparable titers of autologous tier-2 NAbs, but the tier-1 NAb titers elicited by the modified trimers were reduced by 3- to 22-fold depending on the test virus. A comparative analysis showed that reduction in the tier-1 NAb response was greater than that conferred by the stabilizing change used to create the SOSIP.v4.1 trimer. The residual tier-1 NAb responses in the sera from the SOSIP.664-E64K.M1M7 group were not reduced by a competing cyclized BG505 V3 peptide, unlike the sera from the SOSIP.664-immunized rabbits. Thus, the tier-1 NAb response to the modified trimers is not directed at V3 epitopes or at least not at ones that can be mimicked by a cyclized peptide. Other epitopes for tier-1 NAbs, such as those associated with the CD4bs or the CD4-induced coreceptor-binding site, are the most likely targets for the residual tier-1 NAbs. Overall, we conclude that the masking glycans added to V3, alone or together with the E64K substitution, have ablated the immunogenicity of the V3 region on the modified BG505 SOSIP trimers. As the autologous tier-2 NAb titer was not increased, we again find no evidence that V3-dependent tier-1 NAbs interfere with the overall immunogenicity of SOSIP trimers under these experimental conditions in which the autologous tier-2 NAb is a strong and probably immunodominant response (15, 22, 23). These findings are also generally consistent with the observation that ablating the V3 response to an uncleaved gp140 protein in mice did not divert the antibody response to other epitopes on the same immunogen (34).

We consider it possible that off-target non-NAb responses could impede the initiation or evolution of bNAbs driven by germ line-targeting Env proteins, including appropriately designed SOSIP trimers (11, 24, 25, 35, 36). Decreasing the immunodominance of non-NAb epitopes such as those in V3 may be particularly important in this context. Thus, the human antibody repertoire appears to contain an abundant array of germ line precursors for V3 and other non-NAb epitopes, and this subset of naive B cells is readily activated by Env immunogens; once initiated, the non-NAb lineages evolve to acquire high affinity without undergoing extensive or unusual somatic hypermutation (SHM) events (24, 25, 35). The germ line precursors of bNAbs are, however, far rarer, and they are not so easily activated by Env proteins; the initial acquisition of long H3 complementarity-determining regions (CDR) and multiple, and often atypical, SHM events are involved in the bNAb maturation process (24, 25, 35, 37, 38). The higher affinity of germ line non-NAb precursors for Env proteins over their bNAb counterparts may give them a selection advantage within the germinal center (GC), based on the outcome of B cell activation experiments *in vitro* (39). Competition within the GC for the same resources implies that higher-affinity B cell clones may have a selective advantage during the critical early stages of Env protein immunization (25, 37, 39, 40). There is, then, a sound rationale for preventing the activation of high-affinity non-NAb precursors to give their lower-affinity bNAb counterparts a better chance of becoming activated and then undergoing the SHM process (25, 35, 40, 41). Reducing the immunogenicity of V3 and other non-NAb epitopes on germ line-targeting SOSIP trimers, including by using the glycan-masking method that we have described here, may, therefore, be worthwhile.

## MATERIALS AND METHODS

### Env construct design.

The BG505 clade A and B41 clade B SOSIP.664 trimers have been described previously (5, 14). Point substitutions in these *env* genes were made using a QuikChange site-directed mutagenesis kit (Agilent Technologies). Briefly, a primer pair was designed for each mutation or two adjacent mutations that could be accommodated in a single primer without compromising its melting temperature. To allow trimers to be used in SPR and ELISA antigenicity assays, either a GSGSGGSG spacer connected to an eight-histidine (His) tag or a GS spacer followed by a D7324 epitope tag was added immediately C-terminal to residue 664 in the gp41 ectodomain (gp41_ECTO_). From this point, we refer to the presence of the tags only when it is relevant to understanding the experiment. Nontagged versions of trimers were used for rabbit immunizations.

### Env protein expression and trimer purification.

All SOSIP.664 and derivative trimers were produced by transient transfection of 293F cells in serum-free medium, essentially as previously described (5, 13, 14). Env proteins were purified from culture supernatants using affinity columns of bNAb 2G12 or PGT145, which were made using a CNBr-activated Sepharose 4B resin (GE Healthcare) as previously described (5, 13, 14). In each case, the culture supernatant was flowed through the column at a constant rate of 1 ml/min, the beads were washed with buffer (20 mM Tris-HCl, 500 mM NaCl, pH 8), and the Env proteins were eluted with 3 M MgCl_2_ (pH 7.2). The eluted proteins were immediately buffer exchanged into trimer storage buffer (10 mM Tris-HCl, 75 mM NaCl, pH 8) and concentrated using a 100-kDa-cutoff Vivaspin column (GE Healthcare). A Superdex 200 26/60 size exclusion chromatography (SEC) column and the same buffer were then used to isolate trimer fractions, which were pooled, concentrated, and stored at −80°C.

To produce culture supernatants containing unpurified SOSIP.664 Env proteins for pilot-scale assessments by ELISA (see Results), the transfection procedures involved 293T cells, and the culture medium contained 5% fetal bovine serum (FBS).

### BN-PAGE.

The affinity-purified Env proteins or fractions derived from SEC column runs were analyzed on blue native (BN)-PAGE gels (Invitrogen), which were stained with Coomassie blue to visualize protein bands (5, 13, 14).

### Site-specific N-glycosylation analysis.

Approximately 100 μg of the BG505 and B41 SOSIP.664-E64K.M1M7 trimers was reduced and alkylated and then digested in solution using trypsin (Promega, Madison, Wisconsin) as described previously (42). Briefly, trypsin was added to trimers at a 1:30 ratio (wt/wt), and the mixture was incubated for 12 h at 37°C. The resulting glycopeptides were enriched using a ProteoExtract Enrichment kit according to the manufacturer's instructions (Merck Millipore, Darmstadt, Germany). They were then dried, reconstituted in 1% formic acid, and analyzed by reverse-phase liquid chromatography-tandem mass spectrometry (LC-MS/MS) using a Fusion Orbitrap mass spectrometer (Thermo Fisher Scientific, San Jose, CA) coupled to an EASY nLC 1200 system with a PepMap C_18_ column (75 μm by 50 cm). Data interpretation and quantification procedures were performed using Byonic and Byologic software (Protein Metrics, San Carlos, CA), followed by manual assessment (42).

### Antibodies.

Monoclonal antibodies (MAbs) were obtained as gifts or purchased from the following sources: VRC01 (John Mascola); PG9, PG16, PGT122, PGT128, PGT145, PGT151, b6, and b12 (International AIDS Vaccine Initiative); 2G12 (Polymun Scientific); 39F, 17b, 19b, and 14e (James Robinson).

### SPR.

Surface plasmon resonance (SPR) assays were carried out as described previously (21). Briefly, purified His-tagged trimers were captured onto CM5 chips (GE Healthcare) by anti-His antibodies. The anti-His antibodies were covalently immobilized in amounts yielding 15,000 response units (RU). The trimers were then captured to immobilizations levels of 500 RU (equivalent to the ligand response, *R_L_*). Test MAbs were injected at a concentration of 500 nM, with a flow rate of 50 μl/min throughout the association and dissociation phases. Control-channel and 0-analyte subtractions were performed throughout. After each MAb association and dissociation cycle, the antibody-conjugated surface was regenerated by injecting a single pulse of 10 mM glycine (pH 2.0) for 60 s at a flow rate of 30 μl/min.

### ELISA.

Briefly, purified trimers were captured via their His tags onto preblocked Ni-nitrilotriacetic acid (NTA) 96-well plates (Qiagen) by incubation at 0.5 μg/ml for 2 h in Tris-buffered saline (TBS) containing 5% FBS. Culture supernatants from transfected 293T cells, which contain 5% FBS, were diluted 1:4 in TBS before being added to the enzyme-linked immunoabsorbent assay (ELISA) wells. After the capture stage and washing, test MAbs or related reagents were added for 1 h in the same buffer. Bound MAbs were detected using an appropriate horseradish peroxidase (HRP)-conjugated secondary antibody and the 3,3′,5,5′-tetramethylbenzidine (TMB) substrate system with an optical density endpoint at 450 nm (OD_450_) (Bio-Rad). The 50% binding values (50% effective concentration [EC_50_]) for MAb binding were calculated by plotting the nonlinear regression curves using Prism software, version 5.0.

### Negative stain electron microscopy.

Purified trimers were prepared for NS-EM analysis by adsorbing 3 μl of sample onto a glow-discharged, carbon-coated 400-mesh Cu grid. Following blotting, the samples were stained using either 2% (wt/vol) uranyl formate (3 μl of stain for 60 s) or the Nano-W system (3 μl of stain for 7 s, followed by blotting and a second 3 μl of stain for 15 s) (NanoProbes, Inc.). Data collection, processing, and particle analysis methods have been previously described (5, 14, 15).

### Rabbit immunization procedures and ethics statement.

The various rabbit experiments were all approved and carried out in accordance with protocols provided to the Institutional Animal Care and Use Committee (IACUC) at Covance Research Products (CRP), Inc. (Denver, PA), approval number C0080-16. The rabbits were kept, immunized, and bled at Covance in compliance with the Animal Welfare Act and other federal statutes and regulations relating to animals and adhered to the *Guide for the Care and Use of Laboratory Animals* (43).

Rabbit immunizations and blood sampling were carried out under contract at CRP essentially as described previously (3, 15). Female New Zealand White rabbits (5 per group) were immunized intramuscularly three times at weeks 0, 4, and 20, each time with 30 μg of trimers formulated with 75 units of Iscomatrix, a saponin-based adjuvant obtained from CSL, Ltd. (Parkville, Victoria, Australia), via the International AIDS Vaccine Initiative (44).

### Viruses and neutralization assays.

NAbs in rabbit sera were detected and quantified with Env-pseudotyped viruses in a TZM-bl cell assay as described previously (note that TZM-bl cells [catalog number 8129] are derived from the HeLa cell line and supplied by the NIH AIDS Reagents Program) (45). For additional information on this assay and all supporting protocols, see the Los Alamos National Laboratory website (http://www.hiv.lanl.gov/content/nab-reference-strains/html/home.htm). NAb assays were carried out at either Duke University Medical Center (DUMC) (45) or the Weill Cornell Medical College (WCMC) (5, 14). Env-pseudotyped viruses at DUMC were made with the SG3Δenv backbone (46); at WCMC, the NL-Luc-AM vector was used (47). The viruses bore either full-length BG505.T332N (at WCMC) or cytoplasmic-tail-deleted BG505.T332NΔCT (at DUMC) envelope glycoproteins. When the two variants were directly compared, no differences in neutralization sensitivities were observed.

Sera from a subset of time points were also tested at WCMC against three tier-1A Env-pseudotyped viruses, MN and SF162 (clade B) and MW965.27 (clade C), and four heterologous tier-2 viruses and at DUMC against a panel of six tier-1 viruses. An amphotropic murine leukemia virus (MLV) Env-pseudotyped virus was used as a negative control at both sites to determine nonspecific inhibition of infection by rabbit sera. In each assay, all serum dilutions were tested in duplicate. In some experiments, a Cys-cyclized peptide based on the BG505 V3 sequence (CTRPNNNTRKSIRIGPGQAFYATGDIIGDIRQAHC) was used as a soluble competitor for NAbs against tier-1 viruses. The peptide (10 μg/ml) was incubated with serial dilutions of the test sera for 30 min prior to addition of virus and cells according to the standard TZM-bl cell neutralization assay protocol. The negative-control peptide was a scrambled version of the MN V3 sequence, HTGKYTYPTNIAIRGRGNKFRNKKI.

Neutralization was defined as the reduction (percent) of the infectivity obtained in the presence of serum. The serum dilution factors reducing infectivity by 50% were calculated from nonlinear regression fits of a sigmoid function (with the maximum constrained to ≤100% and minimum unconstrained) to the normalized inhibition data by the use of Prism software (GraphPad). For convenience, the resulting reciprocal titers, i.e., 50% inhibitory dilution factors (ID_50_), are referred to simply as “titers.” The NAb titers in the groups of rabbits were compared by two-tailed Mann-Whitney U tests (Prism; GraphPad).

## References

[1] Julien JP, Lee JH, Ozorowski G, Hua Y, Torrents de la Pena A, de Taeye SW, Nieusma T, Cupo A, Yasmeen A, Golabek M, Pugach P, Klasse PJ, Moore JP, Sanders RW, Ward AB, Wilson IA 2015 Design and structure of two HIV-1 clade C SOSIP.664 trimers that increase the arsenal of native-like Env immunogens. Proc Natl Acad Sci U S A 112:11947–11952. doi:10.1073/pnas.1507793112. 26372963 PMC4586835

[2] Julien JP, Cupo A, Sok D, Stanfield RL, Lyumkis D, Deller MC, Klasse PJ, Burton DR, Sanders RW, Moore JP, Ward AB, Wilson IA 2013 Crystal structure of a soluble cleaved HIV-1 envelope trimer. Science 342:1477–1483. doi:10.1126/science.1245625. 24179159 PMC3886632

[3] Klasse PJ, LaBranche CC, Ketas TJ, Ozorowski G, Cupo A, Pugach P, Ringe RP, Golabek M, van Gils MJ, Guttman M, Lee KK, Wilson IA, Butera ST, Ward AB, Montefiori DC, Sanders RW, Moore JP 2016 Sequential and simultaneous immunization of rabbits with HIV-1 envelope glycoprotein SOSIP.664 trimers from clades A, B and C. PLoS Pathog 12:e1005864. doi:10.1371/journal.ppat.1005864. 27627672 PMC5023125

[4] McGuire AT, Hoot S, Dreyer AM, Lippy A, Stuart A, Cohen KW, Jardine J, Menis S, Scheid JF, West AP, Schief WR, Stamatatos L 2013 Engineering HIV envelope protein to activate germline B cell receptors of broadly neutralizing anti-CD4 binding site antibodies. J Exp Med 210:655–663. doi:10.1084/jem.20122824. 23530120 PMC3620356

[5] Sanders RW, Derking R, Cupo A, Julien JP, Yasmeen A, de Val N, Kim HJ, Blattner C, de la Pena AT, Korzun J, Golabek M, de Los Reyes K, Ketas TJ, van Gils MJ, King CR, Wilson IA, Ward AB, Klasse PJ, Moore JP 2013 A next-generation cleaved, soluble HIV-1 Env trimer, BG505 SOSIP.664 gp140, expresses multiple epitopes for broadly neutralizing but not non-neutralizing antibodies. PLoS Pathog 9:e1003618. doi:10.1371/journal.ppat.1003618. 24068931 PMC3777863

[6] Sanders RW, van Gils MJ, Derking R, Sok D, Ketas TJ, Burger JA, Ozorowski G, Cupo A, Simonich C, Goo L, Arendt H, Kim HJ, Lee JH, Pugach P, Williams M, Debnath G, Moldt B, van Breemen MJ, Isik G, Medina-Ramirez M, Back JW, Koff WC, Julien JP, Rakasz EG, Seaman MS, Guttman M, Lee KK, Klasse PJ, LaBranche C, Schief WR, Wilson IA, Overbaugh J, Burton DR, Ward AB, Montefiori DC, Dean H, Moore JP 2015 HIV-1 neutralizing antibodies induced by native-like envelope trimers. Science 349:aac4223. doi:10.1126/science.aac4223. 26089353 PMC4498988

[7] Crooks ET, Tong T, Chakrabarti B, Narayan K, Georgiev IS, Menis S, Huang X, Kulp D, Osawa K, Muranaka J, Stewart-Jones G, Destefano J, O'Dell S, LaBranche C, Robinson JE, Montefiori DC, McKee K, Du SX, Doria-Rose N, Kwong PD, Mascola JR, Zhu P, Schief WR, Wyatt RT, Whalen RG, Binley JM 2015 Vaccine-elicited tier 2 HIV-1 neutralizing antibodies bind to quaternary epitopes involving glycan-deficient patches proximal to the CD4 binding site. PLoS Pathog 11:e1004932. doi:10.1371/journal.ppat.1004932. 26023780 PMC4449185

[8] Tong T, Crooks ET, Osawa K, Binley JM 2012 HIV-1 virus-like particles bearing pure env trimers expose neutralizing epitopes but occlude nonneutralizing epitopes. J Virol 86:3574–3587. doi:10.1128/JVI.06938-11. 22301141 PMC3302546

[9] Pancera M, Zhou T, Druz A, Georgiev IS, Soto C, Gorman J, Huang J, Acharya P, Chuang GY, Ofek G, Stewart-Jones GB, Stuckey J, Bailer RT, Joyce MG, Louder MK, Tumba N, Yang Y, Zhang B, Cohen MS, Haynes BF, Mascola JR, Morris L, Munro JB, Blanchard SC, Mothes W, Connors M, Kwong PD 2014 Structure and immune recognition of trimeric pre-fusion HIV-1 Env. Nature 514:455–461. doi:10.1038/nature13808. 25296255 PMC4348022

[10] Chung NP, Matthews K, Kim HJ, Ketas TJ, Golabek M, de Los Reyes K, Korzun J, Yasmeen A, Sanders RW, Klasse PJ, Wilson IA, Ward AB, Marozsan AJ, Moore JP, Cupo A 2014 Stable 293 T and CHO cell lines expressing cleaved, stable HIV-1 envelope glycoprotein trimers for structural and vaccine studies. Retrovirology 11:33. doi:10.1186/1742-4690-11-33. 24767177 PMC4032163

[11] Sanders RW, Moore JP 2017 Native-like Env trimers as a platform for HIV-1 vaccine design. Immunol Rev 275:161–182. doi:10.1111/imr.12481. 28133806 PMC5299501

[12] Ringe RP, Ozorowski G, Yasmeen A, Cupo A, Cruz Portillo VM, Pugach P, Golabek M, Rantalainen K, Holden LG, Cottrell CA, Wilson IA, Sanders RW, Ward AB, Klasse PJ, Moore JP 5 4 2017 Improving the expression and purification of soluble, recombinant native-like HIV-1 envelope glycoprotein trimers by targeted sequence changes. J Virol doi:10.1128/JVI.00264-17. PMC5446630 28381572

[13] Ringe RP, Yasmeen A, Ozorowski G, Go EP, Pritchard LK, Guttman M, Ketas TA, Cottrell CA, Wilson IA, Sanders RW, Cupo A, Crispin M, Lee KK, Desaire H, Ward AB, Klasse PJ, Moore JP 2015 Influences on the design and purification of soluble, recombinant native-like HIV-1 envelope glycoprotein trimers. J Virol 89:12189–12210. doi:10.1128/JVI.01768-15. 26311893 PMC4645310

[14] Pugach P, Ozorowski G, Cupo A, Ringe R, Yasmeen A, de Val N, Derking R, Kim HJ, Korzun J, Golabek M, de Los Reyes K, Ketas TJ, Julien JP, Burton DR, Wilson IA, Sanders RW, Klasse PJ, Ward AB, Moore JP 2015 A native-like SOSIP.664 trimer based on an HIV-1 subtype B env gene. J Virol 89:3380–3395. doi:10.1128/JVI.03473-14. 25589637 PMC4337520

[15] de Taeye SW, Ozorowski G, Torrents de la Pena A, Guttman M, Julien JP, van den Kerkhof TL, Burger JA, Pritchard LK, Pugach P, Yasmeen A, Crampton J, Hu J, Bontjer I, Torres JL, Arendt H, DeStefano J, Koff WC, Schuitemaker H, Eggink D, Berkhout B, Dean H, LaBranche C, Crotty S, Crispin M, Montefiori DC, Klasse PJ, Lee KK, Moore JP, Wilson IA, Ward AB, Sanders RW 2015 Immunogenicity of stabilized HIV-1 envelope trimers with reduced exposure of non-neutralizing epitopes. Cell 163:1702–1715. doi:10.1016/j.cell.2015.11.056. 26687358 PMC4732737

[16] Kwon YD, Pancera M, Acharya P, Georgiev IS, Crooks ET, Gorman J, Joyce MG, Guttman M, Ma X, Narpala S, Soto C, Terry DS, Yang Y, Zhou T, Ahlsen G, Bailer RT, Chambers M, Chuang GY, Doria-Rose NA, Druz A, Hallen MA, Harned A, Kirys T, Louder MK, O'Dell S, Ofek G, Osawa K, Prabhakaran M, Sastry M, Stewart-Jones GB, Stuckey J, Thomas PV, Tittley T, Williams C, Zhang B, Zhao H, Zhou Z, Donald BR, Lee LK, Zolla-Pazner S, Baxa U, Schon A, Freire E, Shapiro L, Lee KK, Arthos J, Munro JB, Blanchard SC, Mothes W, Binley JM, McDermott AB, Mascola JR, Kwong PD 2015 Crystal structure, conformational fixation and entry-related interactions of mature ligand-free HIV-1 Env. Nat Struct Mol Biol 22:522–531. doi:10.1038/nsmb.3051. 26098315 PMC4706170

[17] Stewart-Jones GB, Soto C, Lemmin T, Chuang GY, Druz A, Kong R, Thomas PV, Wagh K, Zhou T, Behrens AJ, Bylund T, Choi CW, Davison JR, Georgiev IS, Joyce MG, Kwon YD, Pancera M, Taft J, Yang Y, Zhang B, Shivatare SS, Shivatare VS, Lee CC, Wu CY, Bewley CA, Burton DR, Koff WC, Connors M, Crispin M, Baxa U, Korber BT, Wong CH, Mascola JR, Kwong PD 2016 Trimeric HIV-1-Env structures define glycan shields from clades A, B, and G. Cell 165:813–826. doi:10.1016/j.cell.2016.04.010. 27114034 PMC5543418

[18] Harris A, Borgnia MJ, Shi D, Bartesaghi A, He H, Pejchal R, Kang YK, Depetris R, Marozsan AJ, Sanders RW, Klasse PJ, Milne JL, Wilson IA, Olson WC, Moore JP, Subramaniam S 2011 Trimeric HIV-1 glycoprotein gp140 immunogens and native HIV-1 envelope glycoproteins display the same closed and open quaternary molecular architectures. Proc Natl Acad Sci U S A 108:11440–11445. doi:10.1073/pnas.1101414108. 21709254 PMC3136299

[19] Lee JH, Ozorowski G, Ward AB 2016 Cryo-EM structure of a native, fully glycosylated, cleaved HIV-1 envelope trimer. Science 351:1043–1048. doi:10.1126/science.aad2450. 26941313 PMC5001164

[20] Ward AB, Wilson IA 2017 The HIV-1 envelope glycoprotein structure: nailing down a moving target. Immunol Rev 275:21–32. doi:10.1111/imr.12507. 28133813 PMC5300090

[21] Yasmeen A, Ringe R, Derking R, Cupo A, Julien JP, Burton DR, Ward AB, Wilson IA, Sanders RW, Moore JP, Klasse PJ 2014 Differential binding of neutralizing and non-neutralizing antibodies to native-like soluble HIV-1 Env trimers, uncleaved Env proteins, and monomeric subunits. Retrovirology 11:41. doi:10.1186/1742-4690-11-41. 24884783 PMC4067080

[22] Cheng C, Pancera M, Bossert A, Schmidt SD, Chen RE, Chen X, Druz A, Narpala S, Doria-Rose NA, McDermott AB, Kwong PD, Mascola JR 2015 Immunogenicity of a prefusion HIV-1 envelope trimer in complex with a quaternary-structure-specific antibody. J Virol 90:2740–2755. doi:10.1128/JVI.02380-15. 26719262 PMC4810637

[23] Chuang GY, Geng H, Pancera M, Xu K, Cheng C, Acharya P, Chambers M, Druz A, Tsybovsky Y, Wanninger TG, Yang Y, Doria-Rose NA, Georgiev IS, Gorman J, Joyce MG, O'Dell S, Zhou T, McDermott AB, Mascola JR, Kwong PD 8 3 2017 Structure-based design of a soluble prefusion-closed HIV-1-Env trimer with reduced CD4 affinity and improved immunogenicity. J Virol doi:10.1128/JVI.02268-16. PMC5411596 28275193

[24] Havenar-Daughton C, Lee JH, Crotty S 2017 Tfh cells and HIV bnAbs, an immunodominance model of the HIV neutralizing antibody generation problem. Immunol Rev 275:49–61. doi:10.1111/imr.12512. 28133798

[25] McGuire AT, Dreyer AM, Carbonetti S, Lippy A, Glenn J, Scheid JF, Mouquet H, Stamatatos L 2014 Antigen modification regulates competition of broad and narrow neutralizing HIV antibodies. Science 346:1380–1383. doi:10.1126/science.1259206. 25504724 PMC4290850

[26] Garrity RR, Rimmelzwaan G, Minassian A, Tsai WP, Lin G, de Jong JJ, Goudsmit J, Nara PL 1997 Refocusing neutralizing antibody response by targeted dampening of an immunodominant epitope. J Immunol 159:279–289. 9200464

[27] Eggink D, Goff PH, Palese P 2014 Guiding the immune response against influenza virus hemagglutinin toward the conserved stalk domain by hyperglycosylation of the globular head domain. J Virol 88:699–704. doi:10.1128/JVI.02608-13. 24155380 PMC3911724

[28] Sliepen K, van Montfort T, Melchers M, Isik G, Sanders RW 2015 Immunosilencing a highly immunogenic protein trimerization domain. J Biol Chem 290:7436–7442. doi:10.1074/jbc.M114.620534. 25635058 PMC4367253

[29] Behrens AJ, Harvey DJ, Milne E, Cupo A, Kumar A, Zitzmann N, Struwe WB, Moore JP, Crispin M 2017 Molecular architecture of the cleavage-dependent mannose patch on a soluble HIV-1 envelope glycoprotein trimer. J Virol 91:e01894-16. doi:10.1128/JVI.01894-16. 27807235 PMC5215339

[30] Behrens AJ, Crispin M 2017 Structural principles controlling HIV envelope glycosylation. Curr Opin Struct Biol 44:125–133. doi:10.1016/j.sbi.2017.03.008. 28363124 PMC5513759

[31] Munro JB, Gorman J, Ma X, Zhou Z, Arthos J, Burton DR, Koff WC, Courter JR, Smith AB 3rd, Kwong PD, Blanchard SC, Mothes W 2014 Conformational dynamics of single HIV-1 envelope trimers on the surface of native virions. Science 346:759–763. doi:10.1126/science.1254426. 25298114 PMC4304640

[32] Lee JH, Andrabi R, Su CY, Yasmeen A, Julien JP, Kong L, Wu NC, McBride R, Sok D, Pauthner M, Cottrell CA, Nieusma T, Blattner C, Paulson JC, Klasse PJ, Wilson IA, Burton DR, Ward AB 2017 A broadly neutralizing antibody targets the dynamic HIV envelope trimer apex via a long, rigidified, and anionic beta-hairpin structure. Immunity 46:690–702. doi:10.1016/j.immuni.2017.03.017. 28423342 PMC5400778

[33] Hu JK, Crampton JC, Cupo A, Ketas T, van Gils MJ, Sliepen K, de Taeye SW, Sok D, Ozorowski G, Deresa I, Stanfield R, Ward AB, Burton DR, Klasse PJ, Sanders RW, Moore JP, Crotty S 2015 Murine antibody responses to cleaved soluble HIV-1 envelope trimers are highly restricted in specificity. J Virol 89:10383–10398. doi:10.1128/JVI.01653-15. 26246566 PMC4580201

[34] Forsell MN, Soldemo M, Dosenovic P, Wyatt RT, Karlsson MC, Karlsson Hedestam GB 2013 Independent expansion of epitope-specific plasma cell responses upon HIV-1 envelope glycoprotein immunization. J Immunol 191:44–51. doi:10.4049/jimmunol.1203087. 23740950

[35] Stamatatos L, Pancera M, McGuire AT 2017 Germline-targeting immunogens. Immunol Rev 275:203–216. doi:10.1111/imr.12483. 28133796 PMC5741082

[36] Medina-Ramirez M, Garces F, Escolano A, Skog P, Del Moral-Sanchez I, Dosenovic P, Hua Y, McGuire AT, Gitlin AD, Freund NT, Yasmeen A, Behrens AJ, Ozorowski G, de Taeye SW, van den Kerkhof TL, Sliepen K, Blane T, Kootstra NA, van Breemen MJ, Pritchard LK, Stanfield RL, Crispin M, Ward AB, Stamatatos L, Klasse PJ, Moore JP, Nemazee D, Nussenzweig MC, Wilson IA, Sanders RW Design and crystal structure of a native-like HIV-1 envelope trimer that engages multiple broadly neutralizing antibody precursors *in vivo*. J Exp Med, in press. 10.1084/jem.20161160 PMC5584115 28847869

[37] Scharf L, West AP, Sievers SA, Chen C, Jiang S, Gao H, Gray MD, McGuire AT, Scheid JF, Nussenzweig MC, Stamatatos L, Bjorkman PJ 2016 Structural basis for germline antibody recognition of HIV-1 immunogens. eLife 5:e13783. doi:10.7554/eLife.13783. 26997349 PMC4811768

[38] Escolano A, Dosenovic P, Nussenzweig MC 2017 Progress toward active or passive HIV-1 vaccination. J Exp Med 214:3–16. doi:10.1084/jem.20161765. 28003309 PMC5206506

[39] Victora GD, Nussenzweig MC 2012 Germinal centers. Annu Rev Immunol 30:429–457. doi:10.1146/annurev-immunol-020711-075032. 22224772

[40] Zhang Y, Meyer-Hermann M, George LA, Figge MT, Khan M, Goodall M, Young SP, Reynolds A, Falciani F, Waisman A, Notley CA, Ehrenstein MR, Kosco-Vilbois M, Toellner KM 2013 Germinal center B cells govern their own fate via antibody feedback. J Exp Med 210:457–464. doi:10.1084/jem.20120150. 23420879 PMC3600904

[41] McGuire AT, Gray MD, Dosenovic P, Gitlin AD, Freund NT, Petersen J, Correnti C, Johnsen W, Kegel R, Stuart AB, Glenn J, Seaman MS, Schief WR, Strong RK, Nussenzweig MC, Stamatatos L 2016 Specifically modified Env immunogens activate B-cell precursors of broadly neutralizing HIV-1 antibodies in transgenic mice. Nat Commun 7:10618. doi:10.1038/ncomms10618. 26907590 PMC4770077

[42] Behrens AJ, Vasiljevic S, Pritchard LK, Harvey DJ, Andev RS, Krumm SA, Struwe WB, Cupo A, Kumar A, Zitzmann N, Seabright GE, Kramer HB, Spencer DI, Royle L, Lee JH, Klasse PJ, Burton DR, Wilson IA, Ward AB, Sanders RW, Moore JP, Doores KJ, Crispin M 2016 Composition and antigenic effects of individual glycan sites of a trimeric HIV-1 envelope glycoprotein. Cell Rep 14:2695–2706. doi:10.1016/j.celrep.2016.02.058. 26972002 PMC4805854

[43] Committee on the Care and Use of Laboratory Animals of the Institute of Laboratory Animal Resources, Commission on Life Sciences, National Research Council. 1996 Guide for the care and use of laboratory animals. National Academy Press, Washington, DC.

[44] Chung KY, Coyle EM, Jani D, King LR, Bhardwaj R, Fries L, Smith G, Glenn G, Golding H, Khurana S 2015 ISCOMATRIX adjuvant promotes epitope spreading and antibody affinity maturation of influenza A H7N9 virus like particle vaccine that correlate with virus neutralization in humans. Vaccine 33:3953–3962. doi:10.1016/j.vaccine.2015.06.047. 26093202

[45] Montefiori DC 2009 Measuring HIV neutralization in a luciferase reporter gene assay. Methods Mol Biol 485:395–405. doi:10.1007/978-1-59745-170-3_26. 19020839

[46] Wei X, Decker JM, Liu H, Zhang Z, Arani RB, Kilby JM, Saag MS, Wu X, Shaw GM, Kappes JC 2002 Emergence of resistant human immunodeficiency virus type 1 in patients receiving fusion inhibitor (T-20) monotherapy. Antimicrob Agents Chemother 46:1896–1905. doi:10.1128/AAC.46.6.1896-1905.2002. 12019106 PMC127242

[47] Marozsan AJ, Kuhmann SE, Morgan T, Herrera C, Rivera-Troche E, Xu S, Baroudy BM, Strizki J, Moore JP 2005 Generation and properties of a human immunodeficiency virus type 1 isolate resistant to the small molecule CCR5 inhibitor, SCH-417690 (SCH-D). Virology 338:182–199. doi:10.1016/j.virol.2005.04.035. 15935415

[48] Kaplan HA, Welply JK, Lennarz WJ 1987 Oligosaccharyl transferase: the central enzyme in the pathway of glycoprotein assembly. Biochim Biophys Acta 906:161–173. doi:10.1016/0304-4157(87)90010-4. 3297152

[49] van den Kerkhof TL, Feenstra KA, Euler Z, van Gils MJ, Rijsdijk LW, Boeser-Nunnink BD, Heringa J, Schuitemaker H, Sanders RW 2013 HIV-1 envelope glycoprotein signatures that correlate with the development of cross-reactive neutralizing activity. Retrovirology 10:102. doi:10.1186/1742-4690-10-102. 24059682 PMC3849187

[50] Gavel Y, von Heijne G 1990 Sequence differences between glycosylated and non-glycosylated Asn-X-Thr/Ser acceptor sites: implications for protein engineering. Protein Eng 3:433–442. doi:10.1093/protein/3.5.433. 2349213 PMC7529082

